# Preclinical Cognitive Markers of Alzheimer Disease and Early Diagnosis Using Virtual Reality and Artificial Intelligence: Literature Review

**DOI:** 10.2196/62914

**Published:** 2025-01-28

**Authors:** María de la Paz Scribano Parada, Fátima González Palau, Sonia Valladares Rodríguez, Mariano Rincon, Maria José Rico Barroeta, Marta García Rodriguez, Yolanda Bueno Aguado, Ana Herrero Blanco, Estela Díaz-López, Margarita Bachiller Mayoral, Raquel Losada Durán

**Affiliations:** 1 Centro de Neurorrehabilitación González Palau, Córdoba, Argentina; 2 Secretarìa de Investigación, Vicerrectorado de Investigación, Innovación y Posgrado, Universidad Siglo 21, Cordoba, Argentina; 3 Cátedras de Física BIomédica, Facultad de Ciencias Médicas, Universidad Nacional de Córdoba, Córdoba, Argentina; 4 Fundación INTRAS,Valladolid, Spain; 5 Instituto de Neurociencias y Bienestar, Insight 21, Universidad Siglo 21, Cordoba, Argentina; 6 Department of Electronics and Computing, University of Santiago de Compostela, Santiago de Compostela, Spain; 7 Department of Artificial Intelligence, National University of Distance Education, Madrid, Spain

**Keywords:** dementia, Alzheimer disease, mild cognitive impairment, virtual reality, artificial intelligence, early detection, qualitative review, literature review, AI

## Abstract

**Background:**

This review explores the potential of virtual reality (VR) and artificial intelligence (AI) to identify preclinical cognitive markers of Alzheimer disease (AD). By synthesizing recent studies, it aims to advance early diagnostic methods to detect AD before significant symptoms occur.

**Objective:**

Research emphasizes the significance of early detection in AD during the preclinical phase, which does not involve cognitive impairment but nevertheless requires reliable biomarkers. Current biomarkers face challenges, prompting the exploration of cognitive behavior indicators beyond episodic memory.

**Methods:**

Using PRISMA (Preferred Reporting Items for Systematic Reviews and Meta-Analyses) guidelines, we searched Scopus, PubMed, and Google Scholar for studies on neuropsychiatric disorders utilizing conversational data.

**Results:**

Following an analysis of 38 selected articles, we highlight verbal episodic memory as a sensitive preclinical AD marker, with supporting evidence from neuroimaging and genetic profiling. Executive functions precede memory decline, while processing speed is a significant correlate. The potential of VR remains underexplored, and AI algorithms offer a multidimensional approach to early neurocognitive disorder diagnosis.

**Conclusions:**

Emerging technologies like VR and AI show promise for preclinical diagnostics, but thorough validation and regulation for clinical safety and efficacy are necessary. Continued technological advancements are expected to enhance early detection and management of AD.

## Introduction

Nowadays, widespread access to health care systems and changes in living conditions have resulted in an aging population, leading to an increase in the prevalence of neurocognitive disorders (NCD) [1,2]. This phenomenon will lead to a societal change and place an additional burden on health care systems. Therefore, the main challenge at this time lies in the development of therapeutic measures at the pharmacological level that can prevent or halt the progression of Alzheimer disease (AD). However, so far, it has not been possible to find a pharmacological product that meets the safety and efficacy criteria necessary for large-scale use [3-5].

Conversely, numerous studies have shown that factors such as access to higher education, leading a healthy lifestyle, controlling cardiovascular risk factors, and being socially active can have a preventive effect by delaying the onset of symptoms and disease progression. In this context, it is of vital importance to identify emerging markers of AD that allow a diagnosis to be made in its preclinical stage.

The definition of preclinical AD varies, marked by criteria similarities and differences. It signifies the initial stage in the AD continuum, characterized by an extended asymptomatic phase with evidence of AD pathology, yet lacking cognitive, behavioral, or activities of daily living impairment (Table 1). Duration varies (6-10 years), contingent on onset age, and progression to mild cognitive impairment (MCI) hinges on factors like age, sex, and apolipoprotein E status. The complexity arises from not all those meeting preclinical AD criteria progressing to MCI or AD dementia, adding nuances to the predictive analysis [6].

**Table 1.** The AD continuum.^a^

AD continuum	Pathological and anatomical evidence of AD	Behavioral and psychological changes and cognitive impairment	Functional deficit		
IWG-2^b^	Asymptomatic, at risk or presymptomatic	Prodromal	Mild AD dementia	Moderate AD dementia	Severe AD dementia
NIA-AA^c^	Preclinical	MCI^d^ (prodromal AD)	AD with mild dementia	AD with moderate dementia	AD with severe dementia
FDA^e^	Stages 1 and 2 (up to 20 years prior to clinical AD)	Stage 3 (episodic memory, executive function, visuospatial function; disease progression to clinical AD	Stages 4-6 (all domains, in a progressive way)		

a AD: Alzheimer disease.

b IWG: International Working Group.

c NIA-AA: National Institute on Aging—Alzheimer’s Association.

d MCI: mild cognitive impairment.

e FDA: Food and Drug Administration.

In this sense then, early biomarkers are crucial for assessing and monitoring AD. These indicators, recommended by the National Institute on Aging and the Alzheimer’s Association, include the assessment of extracellular amyloid beta (Aβ) and hyperphosphorylated tau protein (p-tau) in the brain [7,8]. The latest guidelines classify neurodegeneration using biomarkers such as amyloid positron emission tomography, Aβ42 levels, tau protein, and neuroimaging techniques. Incorporating these biomarkers aids in the early detection and understanding of AD, aligning with evolving clinical practices and research efforts [9].

However, these biomarkers are expensive and not widely available in much of the world, and their results are not always consistent with the clinical manifestations of the patient, since even in the presence of positive markers, symptoms may not develop or may develop incompletely, and the time gap between the appearance of these biomarkers and the onset of symptoms has not yet been fully characterized.

Since the 1990s, there has been an increasing number of publications aiming to identify cognitive-behavioral markers of the shift from “normal” cognition (NC) to early symptoms of the disease [10-13]. In most studies, episodic memory appears as the domain that is altered in the first instance, reporting variability ranging from 20 [14] to 2 years prior to symptom onset [3,13,15-17].

More recent studies have explored other domains that could be altered earlier, especially visuospatial function and executive functions; in addition, spatial navigation tasks have appeared as a new domain with characteristics that encompasses the 2 previous ones [18-23]. Some studies have proposed to evaluate functional (such as modifications in expansive activities of daily living, changes in mobility patterns) and behavioral aspects, as well as conduct a more thorough analysis of companion reports, and even subjective memory complaints. However, there is little agreement on how to measure them and which ones exhibit true early marker behavior of the disease [24-26]. At this point, it is important to mention that the vast majority of studies are oriented to the diagnosis of NCD due to AD, with only a handful of studies exploring some characteristics of frontotemporal dementias or NCD associated with Parkinson disease, though these tend to have a much lower quality of evidence than studies for AD [27-32].

The most current approaches have incorporated artificial intelligence (AI) and machine learning (ML) tools with the purpose of developing multivariate models that take advantage of these advanced technologies to integrate neuroimaging results, neuropsychological variables, and biomarkers in the early diagnosis of AD [4,9,33-35]. In this context, the use of virtual reality (VR) environments presents a novel and yet underexplored opportunity. So far, they have mainly been used in therapeutic applications, but their potential in the field of diagnostics remains to be investigated [31,32,36-41].

This study aims to review prevalent neurocognitive markers for the preclinical stages of tauopathies, particularly AD. It also explores recent research on VR applications in this context and aims to identify the most evidence-supported tests for assessing valuable cognitive domains.

## Methods

The steps followed for article selection are based on the PRISMA (Preferred Reporting Items for Systematic Reviews and Meta-Analyses) methodology [42], as evidenced in recently published literature reviews on similar topics [43,44]. References were obtained from the search for articles in PubMed and Google Scholar. Articles from the last 10 years were included, although we also included 2 articles published more than 10 years ago because of their importance in the field. In the first instance, the terms used for the search were “pre-dementia Alzheimer’s disease,” “dementia early diagnosis,” “dementia early diagnosis financial,” “benefits of early diagnosis of Alzheimer’s disease cost effectiveness,” “benefits of early diagnosis of Alzheimer’s disease,” and “early diagnosis of Alzheimer’s disease.” However, the articles obtained with these operators corresponded to those with primary treatment objectives or involving exclusively biological measures in different fluids, development of new neuroimaging modalities, or developments of AI and ML algorithms without including neuropsychological parameters. We opted for the search strategy proposed by Bastin et al [12] using the following keywords: ((memory AND longitudinal AND Alzheimer’s disease) AND (prodromal OR conversion OR preclinical)), ((mild cognitive impairment AND (Alzheimer’s disease OR dementia) AND neuropsychology AND (prediction OR longitudinal)), and (Alzheimer’s disease AND conversion AND neuropsychology).

When using these terms, none of the papers yielded results that included VR tools for diagnosis, so the pattern ((mild cognitive impairment AND (Alzheimer’s disease OR dementia) AND neuropsychology AND virtual reality)) was incorporated within the search strategy, finding 13 new papers of which 11 were incorporated, including 2 review papers that are discussed towards the end. Two papers were discarded because they were rehabilitation papers. The terms were used in English for most of the search engines, except for Google Scholar where the same terms were used in Spanish. Articles in both languages were included. As inclusion criteria, we selected those papers that made a longitudinal follow-up or an extensive review either through a systematic review or meta-analysis of the proposed topic. Those papers that could show conversion from NC to MCI or from MCI to dementia were weighted.

Based on this, 58 articles were selected based on the reading of the abstract. Studies conducted in animals, those that pursued primary treatment objectives, those conducted in patients with depression or other psychiatric/psychological comorbidity, those whose primary objective was to develop an AI algorithm, those that only used markers from blood, cerebrospinal fluid, urine, saliva, or other biological markers, and research theses were excluded. After reading the full text, those papers whose primary objective was the development of diagnostic imaging methods unrelated to neurocognitive variables (mainly related to functional magnetic resonance imaging [MRI], volumetry, cortical thickness) and without development of neurocognitive assessment (the tests used were not reported, they only showed raw scores of screening tests) were eliminated, as well as those papers that did not include a control group (MCI or normal aging or healthy controls).

Moreover, the Mendeley functionalities were used for the purpose of consolidating all references drawn from the various databases consulted. Subsequently, an integrated matching tool was used to identify and eliminate duplicates. In the initial screening phase, a preliminary selection of papers was made based on their relevance to the research question. To conduct this screening, the titles and abstracts of the papers were used to ascertain whether any of the 7 exclusion criteria set out in Table 2 were met. In instances where a decision was not immediately evident, the article was designated as a potential candidate for the subsequent phase.

**Table 2.** Summary of the reasons for excluded papers.

Reason for exclusion^a^	Values (n=26+17)
**Screening**	
Did not report empirical data from human participants	12
Only biological markers (eg, blood, urine, cerebrospinal fluid)	4
Depression or another psychiatric comorbidity	3
Primary target: artificial intelligence algorithm development	5
Thesis	2
**Eligibility**	
No inclusion of MCI patients or normal aging or controls trials	5
Primary objective in development of diagnostic imaging methods	6
No neuropsychological assessment	6

a Reasons for excluding papers during the screening (n=26) and eligibility process (n=17).

Subsequently, the full text of all papers that had been deemed eligible following the screening phase was considered. This was considered to be the most appropriate set of papers on the topic, having overcome the exclusion criteria set out in Table 2.

## Results

**Figure 1.**
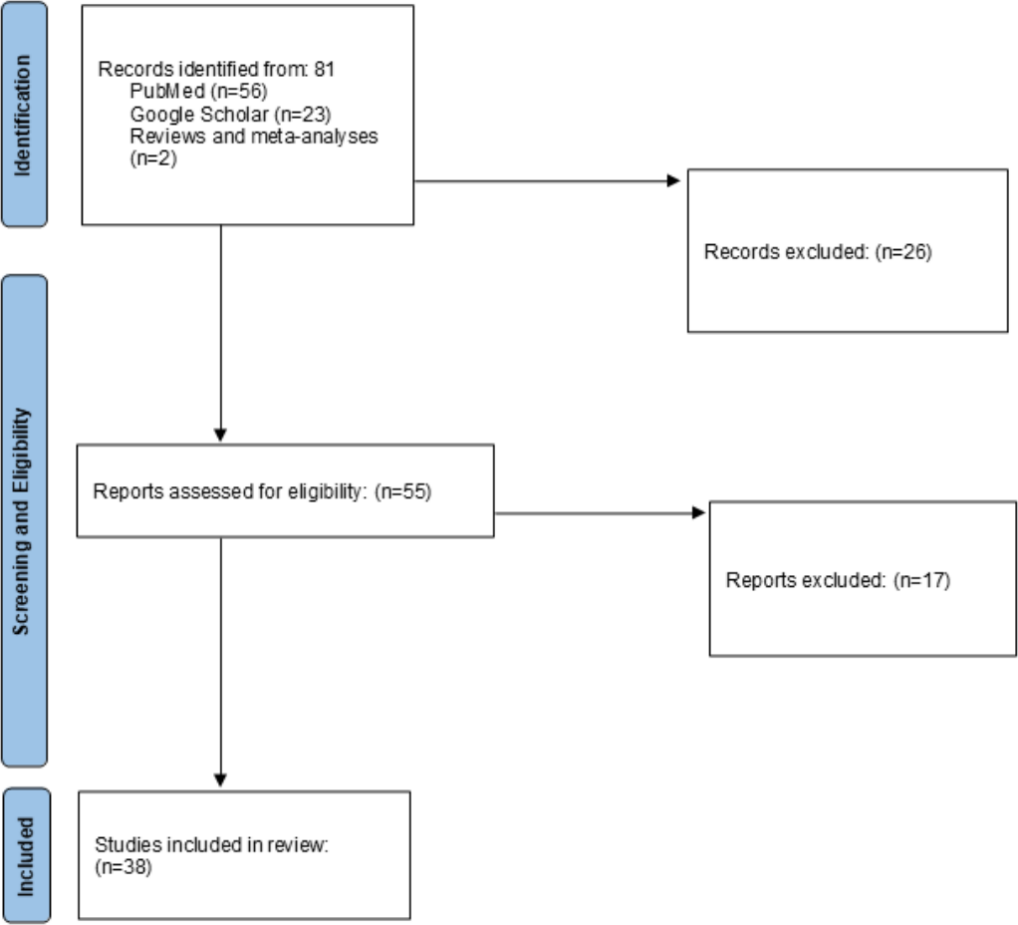
PRISMA (Preferred Reporting Items for Systematic Reviews and Meta-Analyses) flowchart illustrating the process of selecting eligible publications for inclusion in the literature review.

The article selection process is outlined in Figure 1. This narrative outlines key discoveries from selected articles (Table 3 and Table 4)—studies without VR—categorizing the type of work and pinpointing the most early, sensitive, and specific neurocognitive variables according to each paper. Multiple cognitive variables, when presented, are organized by diagnostic profile. The chosen tests for variable measurements are specified. For a more detailed analysis of the reviewed articles, please consult the supplementary material (Multimedia Appendix 1). Moreover, a dedicated section and table are focused on findings related to VR and AI tools utilized for diagnosis (Table 5).

**Table 3.** Synthesis and comparison of selected studies without virtual reality.

	Reference	Neuropsychological domain	Gold standard	Validation
1	[45]	Executive functions	Cognitive markers: Delis–Kaplan Executive Function System Trail Making Test, Stroop, Tower Biological markers: MRI (magnetic resonance image) Cerebrospinal fluid p-tau/amyloid beta 42	CH^c^-pathological amyloid beta 42/tau proteins (n=32) CH-normal amyloid beta 42 Tau proteins (n=33) Mild cognitive impairment (n=39) AD^a^ (n=10)
2	[46]	Episodic memory	Cognitive markers: delayed memory testingBiological markers: atrophy of the hippocampal formation	
3	[47]	Episodic memory	Cognitive markers: Paired-associate immediate recall (Wechsler Memory Scale) Logical memory delayed recall (Story A) (Wechsler Memory Scale) Boston Naming Test (Wechsler Adult Intelligence Scale) Digit Symbol- Substitution Task (Wechsler Adult Intelligence Scale) Biological markers: Cerebrospinal fluid p-tau/amyloid beta 42 Magnetic resonance imaging Blood	Stage 0: n=102 Stage 1: n=46 Stage 2: n=28 SNAP: n=46
4	[48]	Global cognition	Cognitive markers: North American National Adult Reading Test Vocabulary subtest of the Wechsler Adult Intelligence Scale-Revised Years of education Biological markers: Cerebrospinal fluid p-tau/amyloid beta 42 Volume of the right hippocampus Thickness of the right entorhinal cortex Average thickness of seven cortical regions AD-related atrophy	Low CR/^d^normal (n=108) High CR/normal (n=126) Low CR/progressed (n=41) High CR/normal (n=25)
5	[49]	Episodic memory	Cognitive markers: Free and Cued Selective Reminding Test – Free Recall and Total RecallBiological markers: PiB-PET^e^	Aß-positive (n=71) Aß-negative (n=5205)
6	[50]	Episodic memory	Cognitive markers: Free and Cued Selective Reminding Test – Free Recall Logical memory Sequencing task Biological markers: cerebrospinal fluid p-tau/amyloid beta 42	High p-tau/amyloid beta 42 Low p-tau/amyloid beta 42
7	[18]	Episodic memory and spatial navigation memory	Cognitive markers: navigation time, trajectory errors, and delayed recall of a map	Healthy control (n=20) Mild cognitive impairment (n=18) AD (n=20)
8	[51]	Verbal episodic memory	Cognitive markers: Free and Cued Selective Reminding Test Memory Binding Test Rey-Osterreieth Complex Figure Delayed Matched Sample test 48 items Biological markers: PiB-PET^e^	
9	[52]	EEG changes	Cognitive markers: neuropsychological assessmentBiological markers: FDG-PET^f^ PiB-PET^e^ MRI High-density EEG tracing with 256 channels, 1 minute resting closed eyes	
10	[34]	Global cognition	Cognitive markers: Clinical Dementia Rating-Sum of Boxes Alzheimer's Disease Assessment Scale - 11 score Alzheimer's Disease Assessment Scale - 13 score Rey Auditory verbal Learning Test Biological markers: Magnetic resonance imaging Demographic APOE4^b^ genetic	Healthy control (n=184) pMCI^g^ (n=181) sMCI^h^ (n=228) AD (n=192)
11	[35]	Episodic memory	Cognitive markers: Forgetting Index Logical memory Rey Auditory Verbal Learning Test Verbal Paired Associates Learning Mini Mental State Examination ADAS-Cog (Alzheimer´s Disease Assessment Scale- Cognitive) Clinical Dementia Rating Functional Assessment Questionnaire	n=584 409 preserved the mild cognitive impairment diagnosis (4 year follow-up) 175 mild cognitive impairment patients converted to dementia (4 year follow-up)
12	[14]	Visuospatial function and memory task (delayed recall and immediate recall)	Cognitive markers: Complex Figure Test Auditory Verbal Learning Test: Long-Term Memory, Selective Reminding Test, and Total Learning Logical Delayed Recall Memory, Wechsler memory scale-Revised. Inmediate free recall Biological markers: APOE Behavioral Test Score Informants	
13	[9]	Voice	Speech or voice analysis along with analysis of emotional temperature with machine learning algorithms	
14	[30]	Hearing loss	Biological markers: Steady-state Auditory Evoked Potentials P300 Pure tone audiometry	
15	[11]	Global cognition, verbal episodic memory, and attention shifting/flexibility verbal fluency	Cognitive markers: Auditory Verbal Learning Test, Hopkins Verbal Learning Test, Rey Auditory Verbal Learning Test, Logical Memory Mini Mental State Examination Telephone interview for cognitive status Trail Making Test - Part B Semantic verbal fluency test	
16	[53]	Verbal memory task: free delayed recall	Cognitive markers: Rey Auditory Learning Verbal Test	
17	[54]	Global cognition and health	Medical records	AD United Kingdom=20,214 AD France=19,458 Healthy controls United Kingdom=20,214 Healthy controls France=19,458
18	[17]	Sleep parameters	Cognitive markers: Free and Cued Selective Reminding Test Logical Memory Delayed Recall Wechsler Memory Scale - Revised Digit Symbol-Substitution Task - Wechsler Adult Intelligence Scale Mini Mental State Examination Biological markers: Total sleep time, time in non-rapid eye movement sleep, time in rapid eye movement sleep, and sleep efficiency Nonrapid eye movement slow wave activity APOE4 t-tau Amyloid beta 42	n=100
19	[26]	Processing speed, community mobility, driving evaluations, AVD	Cognitive markers: Coding subtest (WAIS-IV) Consortium to Establish a Registry for Alzheimer’s Disease - Semantic Fluency (animals) Controlled Oral Word Association Test Trail Making Test - Part B Timed Instrumental Activities of Daily Living Financial Capacity Instrument-Short Form University of Alabama at Birmingham - Life Space Assessment Useful Field of View Road Sign Test Global Driving Performance Biological markers: MRI-s^i^ Genetic risk alleles (APOE status)	
20	[17]	Sleep disorders	Cognitive markers: Free and Cued Selective Reminding Test Wechsler Memory Scale - Revised: Logical Memory Delayed Recall Digit Symbol-Substitution Task - Wechsler Adult Intelligence Scale Mini Mental State Examination Biological markers: APOE genotype t-tau and amyloid beta 42 in CSF Actigraphy (Actiwatch 2, Philips Respironics) EEG (1 channel)	
21	[19]	Visuospatial working memory, anosognosia, visuomotor control, cognition spatial	Cognitive markers: n-back task and match-to-sample tasks Mental Rotations Test Backward Corsi’s Block-Tapping Test Corsi’s Block-Tapping Test with inhibition Jigsaw-Puzzle Imagery Task Delayed-Response-Activity Test Pathway Span Task Self-Rating Scale of Memory Functions Memory Observation Questionnaire Memory Complaint Questionnaire Metamemory Questionnaire–Ability Subscale Subjective Memory Complaint Questionnaire Anosognosia Rating Scale Clinical Insight Rating Scale Experimenter Rating Scale Tapping and Dotting Subtests (MacQuarrie’s Test for Mechanical Ability) Purdue Pegboard Test Kas’ test Visual-Motor Speed and Precision Test Movement Assessment Battery for Children–Second Edition Eye-Hand Coordination Subtest (Developmental Test of Visual Perception–Third Edition) Ego-Allo Task Four Mountains Test Biological markers: MRI- f Paradigm of resting in PET	
22	[55]	Global cognition	Cognitive markers: Neuropsychiatric Inventory Questionnaire Clinical dementia rating Mini Mental State Examination Dementia Rating Scale Biological markers: Plasma amyloid beta 42, amyloid beta 40, total tau, p-tau181, p-tau231, and neurofilament light Brain autopsy	Low pathology Intermediate ADNC^j^ Intermediate ADNC+ other High ADNC High ADNC+ other Other pathology

a AD: Alzheimer disease.

b APOE: apolipoprotein E.

c CH: Cognitively Healthy

d CR: Cognitve Reserve

e PiB- PET: Pittsburgh - positron emission tomography

f FDG- PET: Fluorodeoxyglucose - positron emission tomography

g pMCI: Progress Mild Congitive Impairment

h sMCI: Stable Mild Cognitive Impairment

i MRI-s: structural magnetic resonance image

j ADCN: AD neuropathological change

**Table 4.** Targeted neuropsychological domain without virtual reality.

Neuropsychological domain	Analytical method	Number	References
Episodic memory	Statistical: 5	5	[18,35,46,47,49]
Global cognition	Statistical: 4; artificial intelligence: 1	5	[11,34,48,54,55]
Verbal episodic memory	Statistical: 2; artificial intelligence: 1	3	[11,51,53]
Visuospatial, navigation	Statistical: 3	3	[14,19,26]
Sleep	Statistical: 2	2	[17,17]
Electroencephalogram	Statistical: 1	1	[52]
Executive functions	Statistical: 1	1	[45]
Voice	Artificial intelligence: 1	1	[9]
Audio	Statistical: 1	1	[30]

**Table 5.** A compilation of selected studies utilizing virtual reality.

	Reference	Type of study	Neuropsychological domain	Gold standard	VR^a^ cognitive tasks
1	[23]	Cross-sectional	Allocentric memory, egocentric memory	VR parks and mazes	Complete neuropsychological assessment; magnetic resonance imaging with volumetry
2	[39]	Cross-sectional	Global cognition	Rivermead Behavioral Memory Test versus Montreal Cognitive Assessment; home selection task; VR versus Montreal Cognitive Assessment	Montreal Cognitive Assessment
3	[24]	Cross-sectional	Functional level in Instrumental Activities of Daily Living	Coffee cup preparation task in a virtual environment	Mini Mental State Examination, Frontal Assessment Battery, Instrumental Activities of Daily Living (Lawton and Brody)
4	[25]	Cross-sectional	Functional level with virtual reality	Tasks of daily life in a virtual environment	Complete neuropsychological battery, Magnetic Resonance Image, cognitive evoked potentials
5	[56]	Cross-sectional	Global cognition	Virtual supermarket	Complete neuropsychological battery
6	[21]	Cross-sectional	Ego/allocentric orientation	VR navigation task	Money’s Road Map test to compare the paper and virtual versions
7	[20]		Ego/allocentric memory	VR navigation task	
8	[57]	Cross-sectional	Navigation task, entorhinal cortex navigation	VR navigation tasks	Digit Symbol, Free and Cued Selective Reminding Test, Mini Mental State Examination, North American National Adult Reading Test, Trail Making Test A and B, magnetic resonance imaging (entorhinal cortex)
9	[58]	Cross-sectional	Comparing traditional paper-based neurocognitive assessment with neurocognitive assessment realized with immersive VR-3D technology.	Precalibrated VR-3D tests, customized 3D-VR testing, traditional 2D digitized tests	3D tasks, 3D virtual maze, 2D tasks, T-maze
10	[59]	Cross-sectional	Prospective memory	Recall of prospective and retrospective components of 7 interventions in a virtual city	Virtual driving: left or right. Two pedals (gas and brake). Control speed.
11	[37]	Cross-sectional	Global cognition, motor performance, cognitive self-report	VR Functional Capacity Assessment Tool	Rey-Osterrieth Complex Figure, Rey Auditory Verbal Learning Test

a VR: virtual reality.

A large majority of articles present verbal episodic memory measured by different tests (Free and Cued Selective Reminding Test, California Verbal Learning Test, Rey Auditory Verbal Learning Test) as the earliest measure with the best sensitivity and specificity ratio for the detection of the preclinical stage of AD and the most recent works propose to support this measure with different neuroimaging formats, genetic profiling (apolipoprotein E), or markers (Aβ, tau, p-tau) in cerebrospinal fluid [11,12,46,49,50,53,60-62]. Among these papers, the one by Gagliardi’s team comparing different neuropsychological tests for the measurement of episodic memory stands out [51].

Harrington and collaborators [45] focused on executive functions using the Stroop test, conducted on individuals without apparent cognitive impairment. They compared those with positive amyloid in cerebrospinal fluid to those without pathology, revealing executive failure preceding memory issues. The study emphasized the absence of standardized protocols for cognitive neural vulnerability in preclinical stages.

The works of Bastin [12] and Gainotti [46] are 2 large review papers that attempted to identify early neuropsychological markers in preclinical and conversion AD, comparing cognitively healthy subjects with and without evidence of pathology. Both papers agreed that episodic memory is the earliest marker, followed by semantic memory, although the protocols used for its assessment differed.

One of the most outstanding articles found in this search is the work of Mistridis et al [63]. The article agrees that verbal episodic memory is an early marker of cognitive impairment, detailing the sequence of decline of various cognitive functions in subjects with NC who progress to MCI. Verbal memory declines about 8 years before MCI, followed by episodic learning, visual memory, and semantic memory about 4 years before MCI. Executive functioning and processing speed declined about 2 years before MCI diagnosis. This suggests that multiple cognitive domains are valuable in assessing preclinical AD.

Another paper that attempted to establish a timeline is that of Soldan et al [47], which used a composite score to evaluate 4 study groups of cognitively unimpaired patients, with the definitions of stage 0 (high Aβ and low tau), stage 1 (low Aβ and low tau), stage 2 (low Aβ and high tau), and suspected non-AD pathology (high Aβ and high tau). The article used linear mixed-effects models to estimate longitudinal cognitive composite scores among individuals in 4 preclinical AD groups. Adjusted for baseline factors, stage 2 individuals showed greater impairment and lower baseline scores compared to others, while stage 0, stage 1, and suspected non-AD pathology groups showed no significant differences. Involving 222 NC adults over 11 years, those in stage 2 (low Aβ, high tau/p-tau) showed markedly lower baseline scores and greater cognitive impairment, suggesting that abnormal amyloid and tau levels are necessary for cognitive differences in NC individuals.

Skills related to visuospatial function, which could prove important in the development of VR environments, were addressed in depth in the work of Caselli et al [14] and Iliardi et al [19]. The first study suggested that visuospatial function deteriorates 20 years before clinical signs of AD, aligning with early pathological changes. It aims to sequence neurocognitive alterations in preclinical stages of AD. The second article addressed the importance of assessing visuospatial working memory, anosognosia, and visuomotor control in patients with MCI, who show worse performance compared to healthy controls and sometimes like patients with dementia. Assessment of visuomotor abilities may help distinguish high-risk AD patients from nonrisk AD patients.

Wadley and colleagues [26] focused on processing speed impact on patient functionality, utilizing an ecological approach integrating neurocognitive and functional variables from professional observations, informant reports, and self-reports. They correlated these with neuroimaging and genetic markers, suggesting processing speed as a stronger correlation of everyday abilities than MRI patterns consistent with AD, and more accessible for measurement. Although neuroimaging demonstrates AD-related neurodegeneration, processing speed captures combined effects, comorbidities, and cognitive reserve, serving as an early marker for functional outcomes.

Among the studies that showed other tools for assessing preclinical stages of NCD that complemented cognitive neural vulnerability, there are studies that incorporate high-resolution electroencephalography measurements [52], olfactory markers [64], auditory [30], retinal thickness measurement by optical coherence tomography [64], use of ML algorithms for speech analysis [9], and home-based sleep studies from specific devices [17]. Some of these works were intended to assess preclinical stages of dementias other than AD, and in the review done by Khan et al in 2020 [9], the proposal of voice analysis in audios and videos allowed for this analysis in several languages. These studies suggest challenges with tests in English, requiring validation for diverse languages and contexts. However, they feature smaller populations, necessitate specific devices, and occur in nonclinical settings.

Literature on the use of VR for early diagnosis of preclinical NCD was limited, unlike its extensive use in cognitive skill training. Papers related to VR for diagnosis or treatment increased from 2008 to 2017, declined until 2020, then started rising again (Table 5).

By 2011, it was confirmed that ego and allocentric memory, linked to the parietal association cortex and medial temporal cortex, could serve as early markers of cognitive decline. VR models, like park or maze navigation, facilitated assessment [23]. Moreover, a Canadian team compared NC subjects with MCI patients using the Montreal Cognitive Assessment, Rivermead Behavioral Memory Test, and a VR test where subjects choose an apartment. The VR test correlated positively with the Montreal Cognitive Assessment, unlike traditional tests, but the sample size was small [39].

The study of Allain et al [24] compares cognitively healthy controls with patients with AD (varying stages, Mini Mental State Examination 18‐26 points) regarding functionality using a virtual coffee preparation task. Measures include completion time, achievement, and error scores compared with real-world tasks. With 24 patients with AD and 32 controls, the study demonstrates VR’s feasibility in studying AD deficits in ecologically valid environments, though limited to a single task.

In 2014, Tarnanas et al [65] conducted research correlating daily living activities assessment in virtual environments with biomarkers like neuropsychological tests, event-related potentials, and MRI. Their study, robust compared to others, suggests VR performance equals biomarkers, attributed to VR’s cognitive fidelity and rich behavioral data reflecting early-stage neurocognitive processes.

Zygiuris et al [56] introduced the virtual supermarket, a VR tool for rehabilitation and screening. The article extensively examined its validity with various measures and set cutoff points for a diagnostic algorithm. Several papers corroborate the apparent sensitivity of visuospatial functions in early NCD detection, evaluating ego and allocentric orientation. Morganti [21] compared a paper and VR version of Money’s “Road Map” test, noting the virtual version’s increased difficulty. They hypothesized that participants solve the traditional version through egocentric spatial transformations, while VR decisions are made based on on-screen interactions. This makes VR tools an interesting alternative, but one that needs to be carefully evaluated to determine what factors influence the results. Similarly, Mohammadi et al [20] used a VR navigation task to distinguish between monodomain and multidomain amnestic MCI, patients with AD, and normal controls, comparing results with traditional neuropsychological tests. They analyzed correct responses and response times in neighborhood and maze environments, establishing distinctive patterns in orientation (ego/allocentric) and visual/verbal memory.

Howett et al [57] published a study correlating entorhinal cortex volume measures, proposed as an early AD marker, with changes in immersive VR tests. They found consistent results with traditional neuropsychological tests, suggesting navigation tasks aid in early AD diagnosis [57]. Within this group, Lecouvey et al [59] utilized VR to evaluate prospective memory through tasks in a virtual city, comparing it with traditional neurocognitive measurements. Results confirmed early prospective memory alterations in AD, suggesting VR as a reliable assessment tool.

Turner’s team [37] created a tablet-based tool evaluating real-world task competence in a realistic VR setting, assessing cognition, motor skills, and self-reported cognitive abilities in patients with Parkinson disease.

To conclude, 2 articles in this review elaborated VR protocols to assess neurocognitive domains, revealing the progression from NC to MCI, focusing on memory and visuospatial functions. In 1 study, an Italian team devised a protocol to detect early cognitive signs of conversion in AD, focusing on egocentric and allocentric spatial representations. Previous studies showed alterations, especially in allocentric frames, in patients with amnesia with MCI and AD. Their innovation lies in proposing an intrinsic connection between egocentric/allocentric frames and spatial relations. The results revealed deficits in allocentric coordinated judgments, implying a deviation towards AD in the representation of metric distances [22]. In the other study, Machado et al [58] highlighted the use of VR environments, in particular 3D mazes, as diagnostic tools for MCI or dementia. Their study compares traditional methods with VR environments, combining precalibrated 3D tests with validated 2D neuropsychological assessments, allowing for assessment across several cognitive domains.

Finally, these studies, when paired with advanced AI algorithms, hold promise for early diagnosis. They enable cross-referencing of various variables, enhancing diagnostic accuracy [4,33,34,57,66].

## Discussion

### Principal Findings

The objective of the Discussion section is to integrate the findings of this literature review and to explore their implications for advancing early diagnostic methods for AD using VR and AI. This section considers the strengths and limitations of current approaches, while also highlighting the potential role of these emerging technologies. Our findings indicate that while verbal episodic memory is a sensitive preclinical marker of AD, other cognitive domains, such as executive function and processing speed, may also serve as valuable early indicators. Furthermore, we examine the significant untapped potential of VR for the assessment of complex cognitive behaviors. VR has the capacity to simulate real-world environments, thereby enabling the evaluation of subtle deficits in cognition that would otherwise be challenging to capture in traditional assessments. AI, with its capacity to analyze multidimensional data, provides a promising approach for integrating biomarkers, cognitive data, and behavioral patterns. Nevertheless, there are considerable obstacles to the clinical implementation of these technologies, particularly in relation to the processes of validation, regulation, and ethical consideration.

The diagnosis of NCD involves advanced pathological definitions based on biomarkers but lacks changes in treatment perspectives. AD remains a paradigm, guiding NCD studies. Although pharmacological treatments are lacking, lifestyle and cardiovascular health modifications may prevent or delay onset, highlighting the importance of early diagnosis for effective intervention.

For more than 30 years, neuropsychological tests have provided the possibility of a more accurate diagnosis and of generating the basis for more specific pharmacological and nonpharmacological treatment plans. However, in recent years, with the advent of biomarkers, the scenario has become more complex [60].

Combining various diagnostic methods like amyloid and tau protein determination in cerebrospinal fluid, positron emission tomography scans with fluorodeoxyglucose or florbetapir, and brain volumetric measurements enhances diagnostic accuracy. However, controversies arise as apparently healthy individuals exhibit positive pathological markers without developing measurable symptoms over extended periods, challenging current diagnostic instruments [6,7,47,67-69].

Incorporating neuropsychological tests into composite diagnostic schemes becomes crucial for reliable conclusions in preclinical disease stages. Verbal episodic memory emerges as the earliest altered neurocognitive domain, followed by visual and semantic memory, despite measurement controversies [3,12-14].

Recent studies suggest executive functions and processing speed alterations as early markers preceding declines in episodic and semantic memory. Improving processing speed through training protocols is vital for maintaining functionality in cognitively demanding tasks like driving and managing finances for individuals with MCI [26,28,31,32,45].

Enhancing diagnostic accuracy for MCI involves indirectly estimating posteromedial parietal cortex function through simple outpatient neuropsychological tasks. Assessing visuomotor abilities in high-risk AD individuals distinguishes conversion probability. Iliardi et al [19] revealed deficits in visuospatial working memory and metamemory as conversion predictors, intricately linked to posteromedial parietal cortex neuronal activity. This innovative approach spans various domains, potentially significantly improving the care and treatment of patients with MCI.

Three recent Spanish review studies focused on identifying cognitive processes assessed via VR and determining commonly used everyday life scenarios in virtual environment design to enhance ecological validity. They noted poor correlation between traditional neuropsychological tests and activities of daily living, prompting the development of technology-based instruments like VR and serious games for evaluation [70-73]. One of these studies reported that 52.3% of works implemented immersive VR, followed by nonimmersive VR (43.2%), and finally semi-immersive VR (4.5%) [70].

VR environments are emerging as new diagnostic tools, but limitations exist. Selected studies were cross-sectional, with few patients evaluated. Gender differences, like navigation strategies in mazes, were noted. Performance in visuospatial tasks is influenced by reference points, instructions, and experimental parameters, highlighting the need for careful consideration in test design [21,24,28,32,56,58].

The translational approach merges animal models and patient evaluations, incorporating complex 3D tasks and standardized neuropsychological tests with automatic analysis, enhancing neuroscience’s cognitive function investigations. A clinical module with preconfigured 2D and 3D tasks simplifies routine patient evaluations [58].

### Limitations of This Literature Review

However, this literature review on preclinical cognitive markers of AD using VR and AI has several notable limitations. First, many studies were conducted with a limited number of participants, which reduces the generalizability of the findings. This small sample size makes it difficult to draw broad conclusions about the effectiveness of VR and AI for early diagnosis in different populations. Second, there is a lack of psychometric validity in some of the cognitive markers used, which raises concerns about the accuracy and reliability of these tools in assessing preclinical stages of AD. In addition, AI algorithms used in early diagnosis are often subject to bias due to the nature of the training data, potentially leading to biased decision-making that could disproportionately affect certain demographic groups. Ethical considerations also remain a challenge, particularly about privacy, consent, and transparency of AI-driven diagnostic processes. Addressing these limitations is critical to the development of robust, fair, and clinically useful AI- and VR-based tools for early detection of AD.

In conclusion, the integration of VR and AI in AD diagnosis represents a rapidly advancing and promising area in medical and neuropsychological research. Key considerations in this domain may include the following aspects:

- Diagnostic accuracy: The fusion of VR and AI provides an avenue for conducting highly accurate and objective assessments of cognitive functions in general, especially visuospatial functions. These tools can identify nuanced alterations in cognitive performance that traditional assessments frequently struggle to detect.
- Early detection: Detecting AD in its preliminary stages is crucial for early intervention and personalized support. VR and AI technologies can detect early signs of cognitive decline before symptoms manifest in daily activities, facilitating more timely and targeted interventions.
- Personalization: Through AI, VR evaluations can be personalized to match the unique capabilities and requirements of each patient. This enables tests to target specific cognitive areas of deficiency, enhancing diagnostic precision and identifying personalized areas for enhancement.
- Greater immersion: VR offers an immersive environment that replicates real-life scenarios and is ideal for evaluating patients’ proficiency in everyday activities like city navigation or shopping. These tasks are challenging to replicate within traditional clinical settings, making VR assessments particularly valuable as they facilitate an ecological and nonintrusive cognitive evaluation of the person.
- Continuous data acquisition: VR and AI facilitate continuous data gathering, enabling precise monitoring of disease advancement and the efficacy of therapeutic measures over time.

### Recommendations for Future Research

Further research is needed to improve the effectiveness of VR and AI practices in early diagnosis of AD, particularly by addressing personalization and diagnostic accuracy. One promising direction is to develop VR assessments that can be tailored to each patient’s unique abilities and needs, ensuring that cognitive assessments are as individualized and responsive as possible. This personalization could contribute to a more accurate understanding of each patient’s cognitive status, thereby improving the sensitivity of early detection. In addition, research should focus on improving diagnostic accuracy by strengthening the psychometric validity of cognitive markers used in VR environments to ensure that these assessments are both reliable and clinically meaningful. In parallel, ongoing efforts are needed to mitigate biases in AI predictions by ensuring that models are trained on diverse datasets that accurately represent the broader population, thereby reducing inequalities in diagnostic outcomes. These future research efforts are essential to refine VR and AI as effective, unbiased, and personalized tools for early detection of AD.

### Conclusions

Preclinical diagnosis of NCD remains challenging, with much more exploration needed. Although VR and AI offer benefits, challenges include expensive hardware, rigorous test validation, and results interpretation by trained professionals. Ethical concerns arise from patient data collection in virtual environments, necessitating strict confidentiality measures. Despite these challenges, VR’s use in AD diagnosis marks noteworthy progress in health care and NCD research. Continued technological advances will improve early detection and management. It is therefore essential to validate and regulate clinical safety and efficacy, delving into new preclinical cognitive markers. Further research is needed to improve the efficacy of VR and AI practices in the early diagnosis of AD, in particular addressing personalization and diagnostic accuracy.

## Supplementary material

10.2196/62914 Multimedia Appendix 1 Tables with the full description of each item and abbreviations.

10.2196/62914 Checklist 1 PRISMA checklist.

## Abbreviations

AD: Alzheimer disease
AI: artificial intelligence
Aβ: amyloid beta
MCI: mild cognitive impairment
ML: machine learning
MRI: magnetic resonance imaging
NC: normal cognition
NCD: neurocognitive disorders
p-tau: hyperphosphorylated tau protein
PRISMA: Preferred Reporting Items for Systematic Reviews and Meta-Analyses
VR: virtual reality
