# Supplementary material for: Preclinical Cognitive Markers of Alzheimer Disease and Early Diagnosis Using Virtual Reality and Artificial Intelligence: Literature Review
Source: JMIR Med Inform. 2025 Jan 28;13:e62914. doi: 10.2196/62914 (PMC11793867; doi:10.2196/62914)
Supplement: Multimedia Appendix 1 [file medinform-v13-e62914-s001.docx]

Table 1. The Alzheimer's disease continuum.

| **ALZHEIMER DISEASE CONTINUNM** | | | | | |
| --- | --- | --- | --- | --- | --- |
|  | Pathological and anatomical evidence of AD | | | | |
|  |  | Behavioral and psychological changes | | | |
|  |  | Cognitive impairment | | | |
|  |  |  | Functional deficit | | |
| IWG-2 | Asymptomatic, at risk or presymptomatic | Prodromal | Mild AD dementia | Moderate AD dementia | Severe AD dementia |
| NIA-AA | PRECLINICAL | MCI  (Prodromal AD) | AD with mild dementia | AD with moderate dementia | AD with severe dementia |
| FDA | Stage 1 & 2 | Stage 3 | Stage 4 | Stage 5 | Stage 6 |
|  |  | Episodic memory, executive function, visuospatial function | All domains (in a progressive way) | | |
|  | Up to 20 years prior Clinical AD | Disease progression  Clinical AD | | | |

Note: According to different clinical classifications: FDA, Food and Drug Administration. IWG, International Working Group. MCI, mild cognitive impairment. NIA-AA, National Institute on Aging—Alzheimer’s Association.

Table 2. Targeted Neuropsychological domain without Virtual Reality

| NEUROPSYCHOLOGICAL DOMAIN | ANALYTICAL METHOD | # | REFERENCES |
| --- | --- | --- | --- |
| Executive functions | Statistical: 1 | 1 | (Harrington et al., 2013), |
| Episodic memory | Statistical: 5 | 5 | (Gainotti et al., 2014), (Soldan et al., 2016), (Papp et al., 2017), (Cogné et al., 2018), (Pereira et al., 2018) |
| Global cognition | Statistical: 4  Artificial Intelligence: 1 | 5 | (Soldan et al., 2017), (Spasov et al., 2019), (Battista et al., 2020), (Nedelec et al., 2022), (Smirnov et al., 2022) |
| Verbal episodic memory | Statistical: 2  Artificial Intelligence: 1 | 3 | (Gagliardi et al., 2019), (Battista et al., 2020), (Ricci et al., 2020) |
| Visuospatial, navigation | Statistical: 3 | 3 | (Caselli et al., 2020), (Wadley et al., 2021), (Ilardi et al., 2022b) |
| EEG | Statistical: 1 | 1 | (Gaubert et al., 2019) |
| Sleep | Statistical: 2 | 2 | (Lucey et al., 2021), (Lucey et al., 2021) |
| Voice | Artificial Intelligence: 1 | 1 | (Khan et al., 2020) |
| Audio | Statistical: 1 | 1 | (Fornaguera Trías & Zamora Córdoba, 2020) |

Table 3. Synthesis and comparison of selected studies without Virtual Reality

|  | REFERENCE | TYPE OF STUDY | NEUROPSYCHOLOGICAL DOMAIN | GOLD STANDARD | VALIDATION | ANALYTICAL METHOD |
| --- | --- | --- | --- | --- | --- | --- |
| 1 | (Harrington et al., 2013) | Transversal, Case Control | Executive functions | Cognitive markers:   - Delis–Kaplan Executive Function System, D-KEFS - (TMT, Stroop, Tower)   Biological markers:   - IRM - CSF pTau/ Aß42 | Cognitive Healthy- Pathological Aß42/Tau proteins (n=32)  Cognitive Healthy- Normal Aß42/  Tau proteins (n=33)  MCI (n=39)  AD (n=10) | Statistical |
| 2 | (Gainotti et al., 2014) | Review | Episodic memory | Cognitive markers: Delayed memory testing  Biological markers: Atrophy of the hippocampal formation |  | Statistical |
| 3 | (Soldan et al., 2016) | Longitudinal | Episodic memory | Cognitive markers:   - Paired Associates immediate recall (WMS) - Logical Memory delayed recall (Story A) (WMS) - Boston Naming (WAIS) - Digit-Symbol Substitution from the (WAIS)   Biological markers:   - CSF pTau/ Aß42 - RMI - Blood | Stage 0: (n = 102)  Stage 1: (n = 46)  Stage 2: (n = 28)  SNAP: (n = 46) | Statistical |
| 4 | (Soldan et al., 2017) | Longitudinal | Global cognition | Cognitive markers:   - National Adult Reading Test - Vocabulary subtest of the WAIS-R - Years of education.   Biological markers:   - CSF pTau/ Aß42 - Volume of the right hippocampus - Thickness of the right entorhinal cortex - Mean thickness of seven cortical regions AD-related atrophy | Low CR/ Normal (n= 108)  High CR/ Normal (n= 126)  Low CR/ progressed (n= 41)  High CR/ Normal (n= 25) | Statistical |
| 5 | (Papp et al., 2017) | Longitudinal | Episodic memory | Cognitive markers: FCSRT FR and TR  Biological markers: [PiB]- PET | Aß + (n = 71)  Aß - (n 5 205) | Statistical |
| 6 | (Schindler et al., 2017) | Longitudinal | Episodic memory | Cognitive markers:   - FCSRT FR - Logical memory - Sequencing task   Biological markers: CSF pTau/ Aß42 | High p-Tau/Aß42  Low p-Tau/Aß42 | Statistical |
| 7 | (Cogné et al., 2018) | Transversal | Episodic memory  Spatial navigation Memory | Cognitive markers: Navigation time, Trajectory errors, and Delayed recall of a map | HC (n=20)  MCI (n=18)  AD (n=20) | Statistical |
| 8 | (Gagliardi et al., 2019) | Longitudinal | Verbal episodic memory | Cognitive markers:   - FCSRT - MBT - ROCF - DMS48   Biological markers: [PiB]- PET |  | Statistical |
| 9 | (Gaubert et al., 2019) | Longitudinal | EEG changes | Cognitive markers: Neuropsychological assessment  Biological markers:   - FDG- PET - [PiB]- PET - IRM - High-density EEG tracing with 256 channels, 1-minute resting closed eyes |  | Statistical |
| 10 | (Spasov et al., 2019) | Transversal | Global cognition | Cognitive markers:   - CDRSB - ADAS11 and ADAS13 - RAVLT   Biological markers:   - MRI - demographic, - APOe4 genetic | HC (n= 184)  pMCI (n= 181)  sMCI (n= 228)  AD (n= 192) | Statistical |
| 11 | (Pereira et al., 2018) | Transversal | Episodic Memory | Cognitive markers:   - Forgetting Index - LM: Logical Memory - RAVLT - VPAL: Verbal Paired-Associate Learning - MMSE: Mini Mental State Examination - ADAS-Cog - CDR - FAQ | n = 584  n = 409 preserved the MCI diagnostic (4-year follow-up)  n = 175 MCI patients converted to dementia (4-year follow-up) | Statistical |
| 12 | (Caselli et al., 2020) | Longitudinal | Visuospatial function  Memory task:  Delayed recall  Immediate recall | Cognitive markers:   - CFT - AVLT: LTM, SRT and total learning - (WMS-R), logical delayed recall memory - Immediate free recall   Biological markers:   - APOE - Behavioral Test Score - Informants |  | Statistical |
| 13 | (Khan et al., 2020) | Review | Voice |  |  | Artificial intelligence:   - Audio/video - Neuroimage |
| 14 | (Fornaguera Trías & Zamora Córdoba, 2020) | Review | Hearing loss | Biological markers:   - Steady-state Auditory Evoked Potentials (SEEPs) - P300 - Pure tone audiometry |  | Statistical |
| 15 | (Battista et al., 2020) | Meta analysis | Global cognition  Verbal episodic memory  Attention  shifting/flexibility  verbal fluency | Cognitive markers:   - AVLT, HVLT, RAVLT, LM - MMSE - TICS - TMT-B - Semantic verbal fluency test |  | Artificial intelligence |
| 16 | (Ricci et al., 2020) | Transversal | Verbal memory task: free delayed recall | Cognitive markers: RALVT |  | Statistical:  Correlation of neuropsychological tests with SPECT  Comparison of CS, MCI, and AD |
| 17 | (Nedelec et al., 2022) | Retrospective | Global cognition and health | Medical Records | AD UK= 20214  AD Fr = 19 458  HC UK= 20214  HC Fr = 19 458 | Statistical |
| 18 | (Lucey et al., 2021) | Longitudinal | Sleep parameters | Cognitive markers:   - FCSRT - Logical Memory Delayed Recall WMS-R, - DSST - WAIS - MMSE   Biological markers:   - total sleep time, time in NREM sleep, time in REM sleep, and sleep efficiency. - NREM SWA - APOe4 - t-tau - Amyloid-b42 | n=100 | Statistical |
| 19 | (Wadley et al., 2021) |  | Processing speed  Community mobility  Driving evaluations  AVD | Cognitive markers:   - Coding subtest (WAIS-IV) - CERAD Semantic Fluency (animals) - COWA - TMT B - UFOV subtest 2 (normalized and reverse coded). - Timed Instrumental - TIADL - FCI- SF - UAB Life Space Assessment - UFOV - Road Sign Test - Global Driving Performance   Biological markers:   - Structural MRI - Genetic risk alleles (APOE status) |  | Statistical |
| 20 | (Lucey et al., 2021) | Longitudinal | Sleep disorders | Cognitive markers:   - FCSRT - WMS-R: Logical Memory Delayed Recall - DSST- WAIS - MMSE   Biological markers:   - APOE Genotype - Total Tau and amyloid-b42 in LCR - Actigraphy (Actiwatch 2, Philips Respironics) - EEG (1 channel) |  | Statistical |
| 21 | (Ilardi et al., 2022b) | Review | Visuospatial working memory  Anosognosia  Visuomotor control  Cognition spatial | Cognitive markers:   - n-back task and match-to-sample tasks - Mental Rotations Test - Backward Corsi’s Block-Tapping Test - Corsi’s Block-Tapping Test with inhibition - Jigsaw-Puzzle Imagery Task - Delayed-Response-Activity Test - Pathway Span Task - SRSMF: Self-Rating Scale of Memory Functions - MOQ: Memory Observation Questionnaire - MAC-Q: Memory Complaint Questionnaire - MMQ-A: Metamemory Questionnaire–Ability Subscale - SMCQ: Subjective Memory Complaint Questionnaire - ARS: Anosognosia Rating Scale - CIRS: Clinical Insight Rating Scale - ERS: Experimenter Rating Scale - Tapping and Dotting Subtests (MacQuarrie’s Test for Mechanical Ability, MTMA) - Purdue Pegboard Test - Kas’ test - VMSPT: Visual-Motor Speed and Precision Test - MABC–2: Movement Assessment Battery for Children–Second Edition - Eye-Hand Coordination Subtest (Developmental Test of - Visual Perception–Third Edition, DTVP-3) - EAT: Ego-Allo Task - 4MT: Four Mountains Test   Biological markers:   - RMN- f - Paradigm of resting in PET |  | Statistical |
| 22 | (Smirnov et al., 2022) | Longitudinal | Global cognition | Cognitive markers:   - NPI: Neuropsychiatric Inventory - CDR: Clinical Dementia Rating - MMSE: Mini-Mental State Examination - DRS: Dementia Rating Scale   Biological markers:   - Plasma Aβ42, Aβ40, total Tau, P-tau181, P-tau231 and neurofilament light (NfL) - Brain autopsy | Low pathology  Intermediate ADNC  Intermediate ADNC + other  High ADNC  High ADNC+ other  Other pathology | Statistical |

Table 4. Synthesis and comparison of selected studies with Virtual Reality

|  | REFERENCE | TYPE OF STUDY | NEURO/  PSYCHOLOGICAL DOMAIN | VIRTUAL COGNITIVE TASK | GOLD STANDARD | OUTCOMES |
| --- | --- | --- | --- | --- | --- | --- |
| 1 | (Weniger et al., 2011b) | Cross-sectional | Allocentric memory  Egocentric memory | RV parks and mazes | Complete Neuropsychological Assessment  MRI with volumetry. | The results indicate that MCI patients had significantly reduced size of the hippocampus bilaterally and of the right-sided precuneus and inferior parietal cortex. |
| 2 | (Nolin et al., 2013) | Cross-sectional | Global Cognition | Home selection tasks | MoCA | He observed a significant positive correlation between the virtual reality task and the MoCA, but not between the traditional task and the MoCA, |
| 3 | (Allain et al., 2014b) | Cross-sectional | Functional level | Coffee cup preparation task in a virtual environment. | MMSE  FAB  IADL (Lawton y Brody) | Attempts to use virtual environment to assess the functional aspect in the definition of dementia and considers information from caregivers for the final result. It does not specify the stage for which it would be useful. |
| 4 | (Tarnanas et al., 2014b) | Cross-sectional | Functional level | Tasks of daily life in a virtual environment | Complete neuropsychological Assessment  IRM  Cognitive evoked potentials | Evaluates 134 patients with MCI and 75 healthy controls. Uses neuropsychological measures and other markers to correlate with the VR task. Performance of VR is comparable to that of biomarkers such as event-related potential and nuclear magnetic resonance imaging |
| 5 | (Zygouris et al., 2015) | Cross-sectional | Global cognition | Virtual supermarket (VSM) | Complete Neuropsychological Assessment | It aims to adapt a resource previously used for diagnostic training. VSM showed a correct classification rate (CCR) of 87.30% in differentiating between patients with MCI and healthy older adults, while it failed to differentiate between MCI subtypes. At the same time, VSM correlates with several known neuropsychological tests. |
| 6 | (Morganti, 2018) | Cross-sectional | Ego/allocentric orientation | RV navigation task | Money’s Road Map Test To compare the paper and virtual versions | They found significant differences between experimental conditions.  The virtual version seems to be more complex to solve. |
| 7 | (Mohammadi et al., 2018) |  | Ego/allocentric memory | RV navigation task |  | We used VR to determine the allocentric and egocentric memory deficits of subjects with single-domain aMCI (aMCISd) and multi-domain aMCII (amMCImd). For this purpose, we introduced an advanced task of to distinguish these deficits in mild Alzheimer's disease (miAD), aMCIsd |
| 8 | (Howett et al., 2019) | Cross-sectional | Navigation task  EC: entorhinal cortex navigation | RV navigation tasks | Digit Symbol  FCSRT  MMSE  NART  TMT A y B  Biological marker: MRI | They perform immersive VR testing, such as a navigation-based measure of entorhinal activity. Behavioral performance correlated with MRI measures of entorhinal cortex volume. was compared to a battery of cognitive tests considered sensitive and specific for early signs of AD |
| 9 | (Machado et al., 2019) | Cross-sectional | Comparing traditional paper-based VNCs with VNCs realized with immersive VR-3D technology | Pre-calibrated RV-3D tests  Customized 3D-VR testing  Traditional 2D digitized tests. | 3D tasks  3D virtual maze  2D tasks  T-maze | VR testing can assess complex 3D tasks and standardized neuropsychological tests combined with automated analysis. |
| 10 | (Lecouvey et al., 2019) | Cross-sectional | Prospective memory | Recall of prospective and retrospective components of 7 interventions in a virtual city. | Virtual driving:  left or right  two pedals (gas and brake)  control speed | PM is affected early in AD. Automatic and controlled retrieval processes are impaired. Demonstrates reliability of VR to assess complex cognitive functions. |
| 11 | (Turner et al., 2021) | Cross-sectional | Global cognition  Motor performance | Virtual Reality Functional Capacity Assessment Tool (VRFCAT-SL) | R-OCFT  RAVLT  Cognitive self-report | Tablet-based instrument that assesses competency to perform real-world tasks in a highly realistic VR environment. |
